# A Curiosum

**Published:** 1887-10

**Authors:** B. L. Cittinski


### A CURIOSUM.

BY DR. B. L. CITTINSKI.

The following is a translation of the petition of the "left hand," addressed to parents and pedagogues, from the pen of a no less distinguished personage than Benjamin Franklin. It was published in a little French almanac, entitled "Etrenne a' l' Humanite," in the year 1787, just a century ago :

"I take the liberty of addressing myself to all the friends of youth, and to beseech them to have compassion upon my misfortune, and help me to conquer the prejudice of which I am the innocent victim.

I am one of two twin sisters of our family. The two eyes in the head do not resemble each other more completely than I and my own sister do.

My sister and I could perfectly agree together if it was not for the partiality of our parents, who favor her to my great humiliation.

From my infancy I was taught to look upon my sister, as if she was of a higher rank than I. My parents allowed me to grow up without any instruction, while they did not spare any cost on the education of my sister. She had professors of writing, drawing, music and other useful and ornamental performances, but if I happened to touch a pencil, a pen or a needle, I was severely reprimanded, and more than once I was even beaten for being clumsy.

It is true that my sister likes my company and does not despise my co-operation occasionally, but always claimes superiority, and only calls upon me when she needs my assistance.

Now ladies and gentlemen, I do not believe that my complaints are dictated by vanity, oh, no ! they have a more serious basis.

My sister and I are charged by our parents with the work of procuring the necessities of life. Now, if some sickness should befall my sister, and make her unable to work (and I tell you in confidence, my sister is subject to cramps, rheumatism, gout and many other ailments), what will become of our family ? Alas ! we shall perish in misery, for I will not be able even to draw up a supplication for obtaining charity. Even for this present petition I have been obliged to use a stranger's hand.

Oh ! how my parents will yet regret having established such an unjust distinction between two sisters who resemble each other so nearly.

Will you be so kind, ladies and gentleman, as to make my parents realize how unjust it is to be so partial in their treatment of their children, and how necessary it is for them to bestow their care and affection upon their offspring in equal measure.

I am, ladies and gentlemen, with the greatest respect, your most humble servant, "THE LEFT HAND."

